# Case Report: *Klebsiella pneumoniae*-associated pyogenic spondylitis and occult intrahepatic cholangiocarcinoma during calculous cholangitis

**DOI:** 10.3389/fmed.2026.1967835

**Published:** 2026-10-07

**Authors:** Zheng Yang, Yuhe Yan, Hui Wang, Hua Zhou, Mengyi Zheng

**Affiliations:** 1 Department of Infectious Diseases, The Second People’s Hospital of Quzhou, Quzhou, Zhejiang, China; 2 Graduate School of Shanxi Medical University, Taiyuan, Shanxi, China; 3 Department of Radiology, Wangdian People’s Hospital, Jiaxing, Zhejiang, China; 4 Department of Rheumatology and Immunology, The Second People’s Hospital of Quzhou, Quzhou, Zhejiang, China

**Keywords:** acute cholangitis, bile duct stones, intrahepatic cholangiocarcinoma, klebsiella pneumoniae, pyogenic spondylitis

## Abstract

**Background:**

*Klebsiella pneumoniae* is a common Gram-negative pathogen in biliary tract infections and may cause deep-seated infection following bacteremia, although vertebral involvement is uncommon. Severe biliary infection and stone disease may also complicate the recognition of coexisting hepatic malignancy.

**Case presentation:**

A 51-year-old woman presented with calculous acute obstructive suppurative cholangitis complicated by septic shock and multiorgan dysfunction. Blood and bile cultures yielded *Klebsiella pneumoniae*, and her condition improved after urgent biliary drainage and antimicrobial therapy. During a subsequent admission, she developed progressive low-back pain. Lumbar magnetic resonance imaging showed abnormalities involving the L3/4 vertebrae and adjacent paravertebral tissues, initially raising suspicion of spinal tuberculosis. Vertebral biopsy demonstrated focal suppurative inflammation, and metagenomic next-generation sequencing of the same tissue detected 33,735 *K. pneumoniae*-specific reads, with a relative abundance of 82.18% and genome coverage of 59.7%, together with multiple hypervirulence-associated determinants. These findings supported a diagnosis of *K. pneumoniae*-associated pyogenic spondylitis. Meanwhile, serial hepatobiliary imaging showed regression of the right hepatic abnormalities as the infection improved, whereas a focal lesion in the left liver persisted. Subsequent imaging raised suspicion of cholangiocarcinoma, and histopathologic examination after surgical resection confirmed poorly differentiated intrahepatic cholangiocarcinoma.

**Conclusion:**

This case underscores the importance of maintaining diagnostic vigilance even after an initial diagnosis has been established. New focal symptoms or abnormalities that persist or follow a different clinical course should not be automatically attributed to the original disease process, and the possibility of additional coexisting diagnoses should remain under consideration.

## Introduction

Acute cholangitis develops when bacterial infection occurs in an obstructed biliary system, with bile duct stones among the most common underlying causes (1). In moderate-to-severe disease, timely biliary decompression together with antimicrobial and supportive treatment is essential (2). Bile cultures from patients with suspected acute cholangitis commonly yield enteric organisms, with *Enterococcus spp.*, *Klebsiella spp*., and *Escherichia coli* among the organisms frequently isolated (3).

Although bacteremia may extend an initially localized infection beyond the biliary tract, vertebral infection caused by *Klebsiella pneumoniae* is uncommon. *K. pneumoniae* accounts for only a small proportion of pyogenic spondylitis, while virulence-associated features have been reported among isolates from affected patients (4). Recognition may be particularly difficult when spinal symptoms become apparent only after the acute infection has improved or when imaging initially suggests another diagnosis.

Biliary stone disease may also complicate the recognition of coexisting hepatobiliary disease. Recurrent pyogenic cholangitis is associated with an increased risk of cholangiocarcinoma (5), and concomitant cholangiocarcinoma may be difficult to recognize in patients with hepatolithiasis and biliary infection (6, 7). We report a patient with calculous acute cholangitis and *K. pneumoniae* bacteremia who subsequently developed *K. pneumoniae*-associated pyogenic spondylitis and was also found to have occult intrahepatic cholangiocarcinoma. This case emphasizes that, even when an initial infectious diagnosis is well established, new symptoms or persistent abnormalities should not automatically be attributed to the same disease process, as additional diagnoses may coexist.

## Case presentation

A 51-year-old woman with a remote history of bronchitis but no major chronic comorbidities was admitted on April 2, 2026, with progressive jaundice, fatigue, and poor appetite. She did not smoke or drink alcohol, had no known drug or food allergies, and had no relevant family history. Abdominal CT showed multiple left intrahepatic and common bile duct stones with biliary dilatation and gallbladder enlargement. The initial abdominal CT and subsequent serial hepatobiliary imaging findings are shown in Figure 1. She developed septic shock with hepatic, renal, and coagulation dysfunction and was diagnosed with calculous acute obstructive suppurative cholangitis. Because the patient had septic shock and multiorgan dysfunction, definitive surgery was considered high risk. Ultrasound-guided percutaneous transhepatic gallbladder drainage was therefore performed for biliary decompression, infection control, and bile culture. Blood and bile cultures both yielded *K. pneumoniae*. Her infection and cholestasis subsequently improved, and she was discharged on April 21 with the drainage catheter in place.

**Figure 1:**
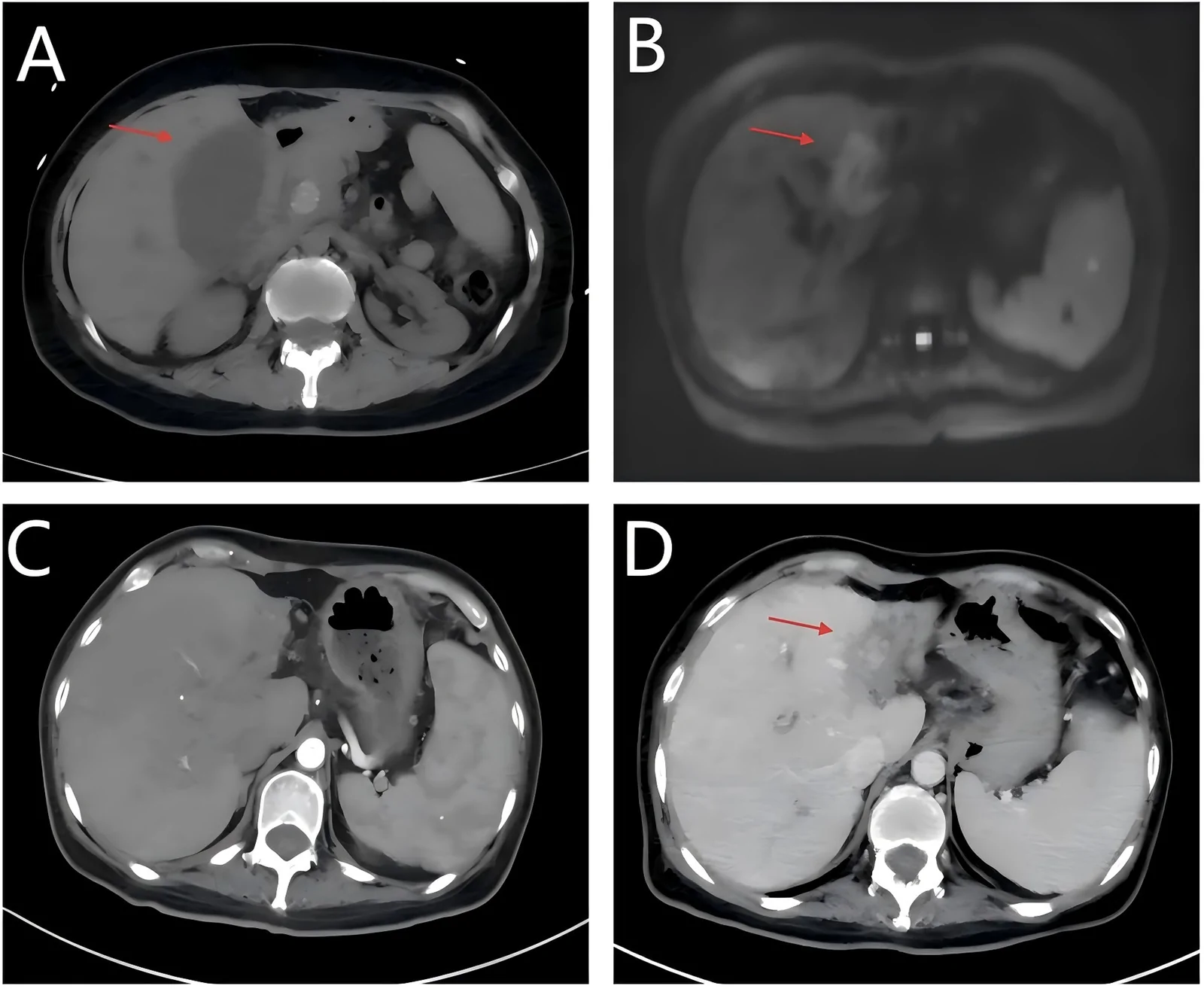
Serial hepatic imaging. **(A)** Abdominal CT obtained on April 2, 2026. Retrospective review showed a subtle low-attenuation lesion in the left hepatic region corresponding to the subsequently diagnosed tumor. **(B)** Diffusion-weighted MRI on May 16 showed regression of the right hepatic infection-related abnormality, whereas the left hepatic lesion remained visible. **(C)** Contrast-enhanced CT on May 26 showed a residual perfusion-related abnormality in the right liver and persistence of the left hepatic lesion. **(D)** Contrast-enhanced CT on July 6 demonstrated a more clearly defined left hepatic mass with abnormalities of the left intrahepatic bile ducts, raising suspicion of cholangiocarcinoma.

She was readmitted on May 15 for further management of the biliary stones and developed progressive low-back pain with lumbar tenderness. Lumbar MRI on May 19 showed abnormalities involving the L3/4 vertebrae and adjacent paravertebral tissues, with psoas involvement. Spinal tuberculosis with paravertebral abscess formation was initially considered, although T-SPOT.TB was negative. Image-guided drainage of the suspected paravertebral abscess was performed on May 25, but no appreciable fluid was obtained. Antimicrobial treatment was continued while further diagnostic evaluation was pursued.

An L4 vertebral biopsy on June 5 showed inflammatory-cell infiltration with focal suppurative changes. A portion of the vertebral-tissue specimen was subjected to DNA-based metagenomic next-generation sequencing (mNGS; PIseq®, WillingMed, Hangzhou, China). Host nucleic acids were depleted using the PathoXtract workflow, followed by unbiased shotgun metagenomic sequencing and bioinformatic pathogen identification. The analysis detected 33,735 *K. pneumoniae*-specific reads, with a relative abundance of 82.18% and genome coverage of 59.7%. Virulence-associated determinants included *iucA-D, iutA, iroB-D*, *iroN, rmpA*, and *rmpA2*. In combination with the preceding *K. pneumoniae* bacteremia, spinal imaging, and histopathologic findings, these results supported the diagnosis of *K. pneumoniae*-associated pyogenic spondylitis. Contrast-enhanced lumbar MRI on June 17 further demonstrated L3/4 vertebral destruction with surrounding inflammatory changes. The spinal imaging, vertebral histopathologic findings, and mNGS results are summarized in Figure 2.

**Figure 2:**
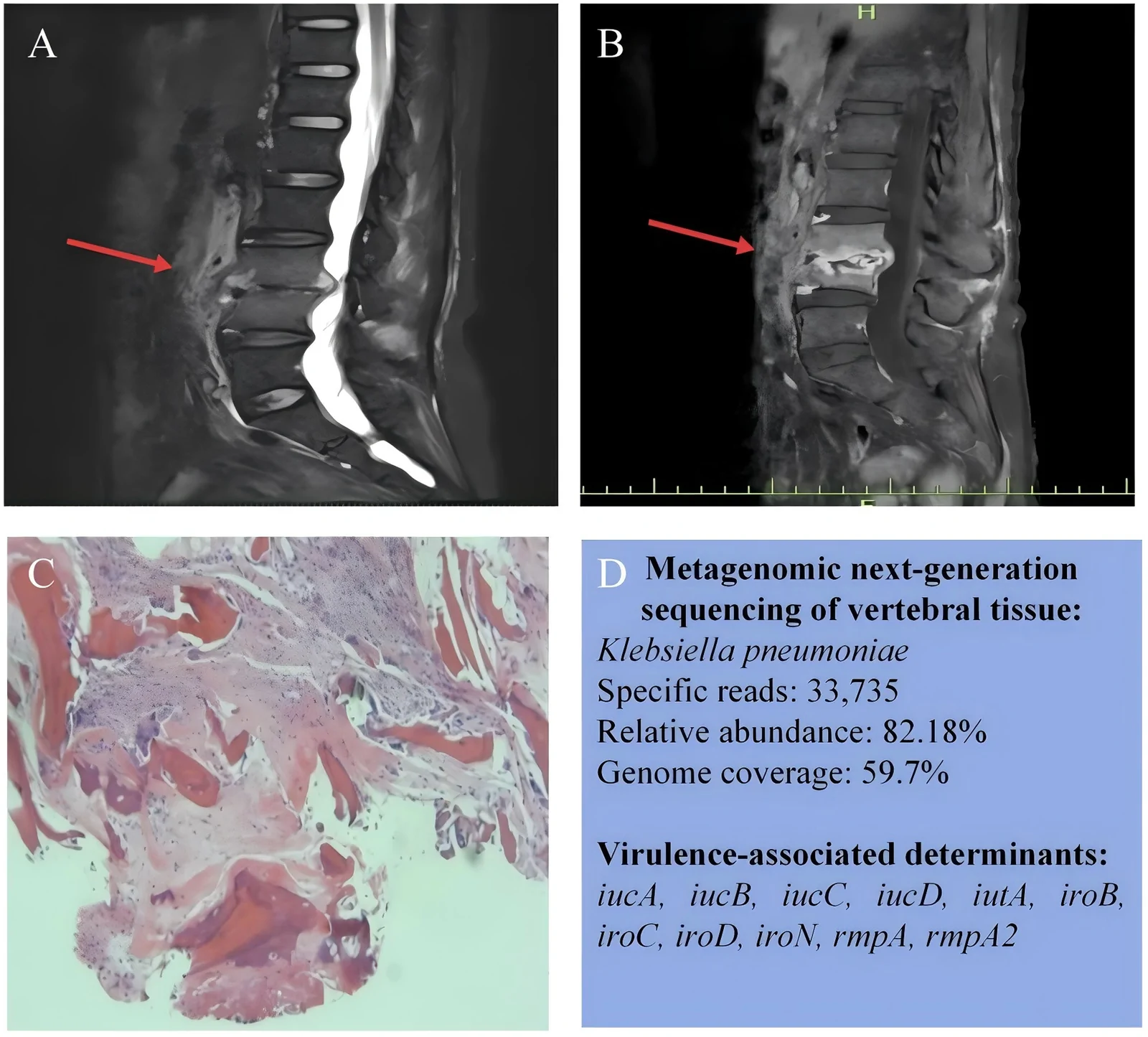
*K. pneumoniae*-associated pyogenic spondylitis. **(A)** Lumbar MRI obtained on May 19, 2026, showing abnormalities involving the L3/4 vertebrae and adjacent paravertebral tissues, with associated psoas muscle swelling. **(B)** Contrast-enhanced lumbar MRI on June 17 showed vertebral destruction and surrounding inflammatory changes. **(C)** Hematoxylin and eosin staining of the L4 vertebral biopsy obtained on June 5 showed inflammatory-cell infiltration with focal suppurative changes. **(D)** Metagenomic next-generation sequencing of vertebral tissue detected *K. pneumoniae*, with 33,735 specific reads, a relative abundance of 82.18%, and genome coverage of 59.7%. Virulence-associated determinants included *iucA-D, iutA, iroB-D, iroN, rmpA*, and *rmpA2*.

During the same clinical course, serial hepatobiliary imaging showed different evolution of the hepatic abnormalities. Serum CA19-9 decreased from 23,509.32 U/mL during the initial admission to 88.15 U/mL after biliary drainage and improvement of the infection. A right hepatic abnormality regressed, whereas a focal lesion in the left liver persisted. Retrospective review of the initial CT showed a subtle abnormality at the same site. Repeat contrast-enhanced CT on July 6 demonstrated a more clearly defined left hepatic mass with abnormalities of the adjacent intrahepatic bile ducts, raising suspicion of cholangiocarcinoma.

Frozen-section examination on July 10 showed poorly differentiated carcinoma favoring cholangiocarcinoma. The patient subsequently underwent left hemihepatectomy, abdominal lymph-node dissection, cholecystectomy, adhesiolysis, common bile duct exploration with stone extraction, choledochoscopy, and T-tube drainage. Histopathologic examination of the resected specimen showed a 5.5 × 5.0 × 3.5 cm poorly differentiated adenocarcinoma, favoring large-duct type intrahepatic cholangiocarcinoma. Representative histopathologic findings are shown in Figure 3. Vascular invasion and perineural invasion were present, the resection margin was negative, and metastatic carcinoma was identified in 1 of 3 examined lymph nodes. The pathological stage was pT2N1Mx. A dedicated long-term follow-up lumbar MRI was not available, and detailed subsequent antimicrobial and oncologic treatment records could not be obtained because further care was provided at another institution. The overall clinical timeline is summarized in Table 1.

**Figure 3:**
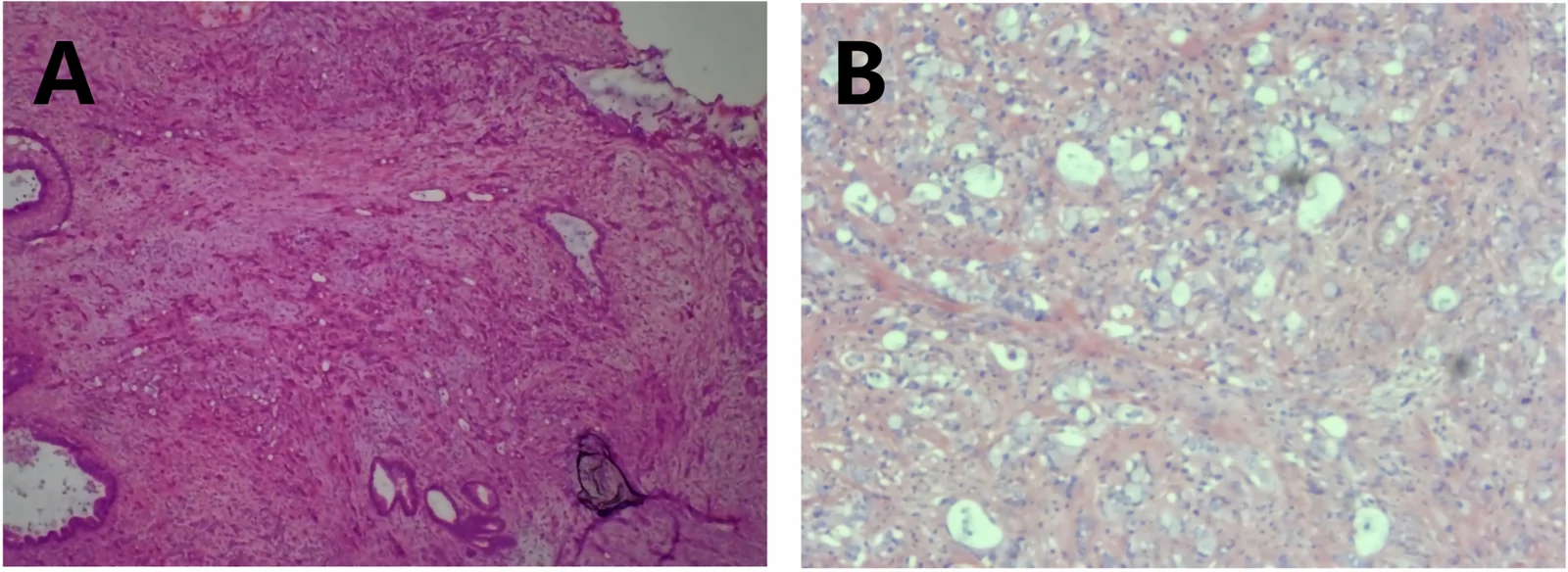
Histopathology of intrahepatic cholangiocarcinoma. **(A)** Intraoperative frozen-section H&E staining obtained during surgery on July 10, 2026, showing malignant histologic features. **(B)** Permanent H&E section of the definitive resection specimen obtained on July 13, showing poorly differentiated intrahepatic cholangiocarcinoma.

**Table 1:** Clinical timeline of the diagnostic and therapeutic course.

Date	Major clinical findings/events	Clinical interpretation and management
2-Apr-26	Progressive jaundice; intrahepatic and common bile duct stones with biliary dilatation; septic shock and multiorgan dysfunction. Blood and bile cultures yielded *K. pneumoniae*.	Calculous AOSC with biliary-source *K. pneumoniae* bacteremia. Urgent percutaneous cholecystostomy performed and intravenous meropenem initiated on April 2.
April 6–21, 2026	Infection and cholestasis markedly improved; CRP and bilirubin decreased substantially.	Antimicrobial therapy was switched to cefoperazone/sulbactam on April 6 and continued until discharge on April 21.
May 15–19, 2026	Readmitted for biliary-stone management; progressive low-back pain and recurrent elevation of inflammatory markers. Lumbar MRI showed L3/4 vertebral and paravertebral abnormalities with psoas involvement.	Spinal tuberculosis/paravertebral abscess initially considered; T-SPOT.TB was negative and multidisciplinary evaluation was undertaken.
May 16–26, 2026	Right hepatic abnormality regressed on serial imaging, whereas a focal left hepatic lesion persisted. CA19-9 decreased from 23,509.32 to 88.15 U/mL after biliary decompression.	Right hepatic findings considered predominantly infection related; persistent left hepatic lesion remained under evaluation.
May 25–June 2, 2026	Image-guided puncture of the suspected paravertebral lesion yielded no fluid; low-back pain and inflammatory activity persisted.	Treated with piperacillin/tazobactam followed by meropenem during this admission. The exact switch date and agent-specific treatment durations were unavailable.
5-Jun-26	L4 vertebral biopsy showed focal suppurative inflammation. Vertebral-tissue mNGS detected 33,735 *K. pneumoniae*-specific reads (82.18% relative abundance; 59.7% genome coverage).	*K. pneumoniae-*associated pyogenic spondylitis diagnosed. Complete subsequent antimicrobial treatment records were unavailable because further care was provided at another institution.
17-Jun-26	Contrast-enhanced lumbar MRI showed L3/4 vertebral destruction with surrounding inflammatory changes.	Imaging further supported pyogenic spondylitis.
July 6–13, 2026	Persistent left hepatic mass became suspicious for cholangiocarcinoma. Frozen section on July 10 favored cholangiocarcinoma; definitive pathology showed poorly differentiated intrahepatic cholangiocarcinoma, large-duct type favored, pT2N1.	Definitive hepatobiliary surgery was performed, including left hemihepatectomy, lymph-node dissection, cholecystectomy, common bile duct exploration with stone extraction, choledochoscopy, and T-tube drainage.
July 23–24, 2026	T-tube cholangiography showed no obvious residual distal common bile duct stone and patent drainage into the duodenum. Abdominal CT showed postoperative changes and persistent L3/4 vertebral/paravertebral abnormalities.	Early postoperative recovery; dedicated long-term spinal imaging follow-up was unavailable.

## Discussion

This case illustrates how a clinically plausible initial diagnosis may shape the interpretation of findings that emerge later in the disease course. Severe calculous cholangitis with *K. pneumoniae* bacteremia provided a coherent explanation for the patient's initial presentation. The subsequent spinal infection was most plausibly related to the preceding bacteremia, whereas the persistent hepatic lesion represented a separate coexisting disease. The latter was particularly difficult to recognize because hepatic abnormalities detected during severe biliary infection could reasonably have been attributed to inflammation. This case also illustrates how anchoring bias may contribute to missed or delayed diagnosis when new findings are interpreted in light of an initial diagnosis rather than prompting reconsideration of alternative explanations (8, 9).

*K. pneumoniae* is an uncommon cause of pyogenic spondylitis, although virulence-associated characteristics have been reported among isolates from affected patients (4). Metastatic spread following bacteremia is a feature of invasive *K. pneumoniae* infection, particularly in infections caused by hypervirulent strains (10). In this patient, *K. pneumoniae* had been isolated from both blood and bile during the initial episode of cholangitis, and progressive low-back pain subsequently developed after the acute biliary infection had improved. Lumbar imaging demonstrated L3/4 vertebral and paravertebral abnormalities, while vertebral biopsy showed focal suppurative inflammation. mNGS of the same tissue detected abundant *K. pneumoniae* sequences, providing additional microbiological evidence for *K. pneumoniae*-associated pyogenic spondylitis. Taken together, the temporal sequence and microbiological, imaging, and histopathologic findings make hematogenous dissemination from the preceding bacteremia the most plausible explanation, although this cannot be confirmed because the original blood and bile isolates were not retained for genomic comparison. The vertebral-tissue mNGS also detected several virulence-associated determinants. Among these, *iucA, iroB, rmpA*, and *rmpA2* have been reported as biomarkers associated with hypervirulent *K. pneumoniae* isolates (11, 12), together with additional genes belonging to the aerobactin and salmochelin systems in the present sample. These findings indicate the presence of virulence-associated genes but do not confirm their functional expression or a hypervirulent phenotype. Because they were detected directly from metagenomic DNA rather than from a cultured isolate, definitive phenotypic confirmation was not possible. The patient had also received prolonged antimicrobial treatment before vertebral biopsy, which may reduce the yield of conventional microbiological culture (13). In this setting, mNGS may provide complementary pathogen detection in suspected spinal infection, including after prior antimicrobial exposure (14, 15). Current IDSA guidelines recommend 6 weeks of parenteral or highly bioavailable oral antimicrobial therapy for most patients with bacterial native vertebral osteomyelitis (16). However, the complete subsequent antimicrobial course in this patient was unavailable because further care was provided at another institution.

The hepatobiliary findings raised a different diagnostic issue. The patient initially had a highly plausible and common clinical sequence of bile duct stones, biliary obstruction, acute cholangitis, and *K. pneumoniae* infection. Against this background, hepatic abnormalities identified during the acute phase could reasonably have been interpreted as infection related. Acute cholangitis can produce transient or heterogeneous hepatic enhancement that may decrease after effective treatment (17, 18), consistent with the right hepatic abnormality that regressed as the infection improved. The left hepatic lesion, however, persisted on serial imaging and was retrospectively visible on the initial CT. This distinction became clinically important because intrahepatic cholangiocarcinoma may occasionally mimic an abscess or other inflammatory lesion (19), and the diagnosis may be particularly difficult in patients with intrahepatic bile duct stones and associated infection (6, 7). More recent longitudinal data have also confirmed an increased risk of subsequent intrahepatic cholangiocarcinoma in patients with hepatolithiasis (20). The marked decrease in CA19-9 from 23,509.32 to 88.15 U/mL after biliary drainage further illustrates why the malignancy was not immediately apparent. Biliary obstruction and inflammation can cause substantial elevations in CA19-9, with levels often decreasing after decompression (21), whereas persistent elevation after relief of obstruction is more frequently observed in malignant pancreatobiliary disease (22). In this patient, however, neither the initial elevation nor its subsequent decline was sufficient to establish or exclude malignancy. The decisive clue was the different evolution of the hepatic abnormalities: the infection-related lesion regressed, whereas the left hepatic lesion persisted and was ultimately confirmed histologically as intrahepatic cholangiocarcinoma. The presence of regional lymph-node metastasis, vascular invasion, and perineural invasion indicated a substantial risk of postoperative recurrence. These pathological findings support the need for postoperative oncologic assessment and consideration of adjuvant therapy; however, detailed subsequent oncologic treatment and long-term outcomes were unavailable because the patient continued care at another institution.

Several limitations should be acknowledged. The original *K. pneumoniae* isolates were unavailable for genomic comparison, preventing confirmation of clonality between the initial bacteremia and the vertebral infection. In addition, long-term dedicated spinal imaging and detailed subsequent treatment records were unavailable because the patient continued care at another institution, limiting assessment of the longer-term response of the spondylitis and oncologic outcome. The key learning points from this case are that new focal symptoms after bacteremia should prompt evaluation for metastatic infection, whereas persistent abnormalities that evolve differently from infection-related lesions should be investigated independently for coexisting disease.

## Patient perspective

I understood that the liver cancer was difficult to recognize during the early stage of my illness because the doctors were treating my severe biliary infection at that time. I am grateful to the medical teams for the timely treatment of the cholangitis and spinal infection and for helping me recover through this difficult period.

## Data Availability

Data may be made available from the corresponding author upon reasonable request and with appropriate institutional approval.
